# Improved Sensing Capability of Integrated Semiconducting Metal Oxide Gas Sensor Devices †

**DOI:** 10.3390/s19020374

**Published:** 2019-01-17

**Authors:** Ayoub Lahlalia, Olivier Le Neel, Ravi Shankar, Siegfried Selberherr, Lado Filipovic

**Affiliations:** 1 Institute for Microelectronics, TU Wien, 1040 Vienna, Austria; selberherr@iue.tuwien.ac.at (S.S.); filipovic@iue.tuwien.ac.at (L.F.); 2 STMicroelectronics Pte Ltd., Singapore 569508, Singapore; olivier.leneel@st.com (O.L.N.); ravi.shankar@st.com (R.S.)

**Keywords:** semiconducting metal oxide, gas sensor, microheater, electro-thermal-mechanical simulation, smartphone, ultra-low power

## Abstract

Semiconducting metal oxide (SMO) gas sensors were designed, fabricated, and characterized in terms of their sensing capability and the thermo-mechanical behavior of the micro-hotplate. The sensors demonstrate high sensitivity at low concentrations of volatile organic compounds (VOCs) at a low power consumption of 10.5 mW. In addition, the sensors realize fast response and recovery times of 20 s and 2.3 min, respectively. To further improve the baseline stability and sensing response characteristics at low power consumption, a novel sensor is conceived of and proposed. Tantalum aluminum (TaAl) is used as a microheater, whereas Pt-doped SnO_2_ is used as a thin film sensing layer. Both layers were deposited on top of a porous silicon nitride membrane. In this paper, two designs are characterized by simulations and experimental measurements, and the results are comparatively reported. Simultaneously, the impact of a heat pulsing mode and rubber smartphone cases on the sensing performance of the gas sensor are highlighted.

## 1. Introduction

Recently, the desire for SMO gas sensors suitable for integration in wearable devices has been particularly strong. This interest is principally driven by the increased market size of gas sensors for consumer applications [1] and the growing concern about the detrimental impact of outdoor air pollution. In 2018, the World Health Organization (WHO) reported that ambient air pollution has caused around 4.2 million deaths, whereas household air pollution has caused about 3.8 million deaths in 2016 alone [2]. However, in order to minimize or eliminate the use of products and processes that pollute our surroundings, especially in the home, one part of the solution includes raising awareness about the quality of the air we breathe and, ultimately, how vulnerable we are to the impacts of climate change. This can be done by making SMO gas sensors available to everyone through their integration with handheld devices such as smartphones and wristwatches, which allow for easy monitoring of the air quality at any time and from any location.

The development of novel gas sensors based on metal oxide films dedicated to wearable devices is still in progress, and the need for reliable sensors with a high selectivity and ultra-low power consumption is one of the most challenging issues burdening SMO gas sensor development and their broad availability. Several limitations and drawbacks must still be overcome before a monolithic integration of the gas sensor with portable devices can be realized:

- A further decrease in the power consumption of SMO gas sensors, operating in DC mode, is necessary to address some innovative and integrated applications. Until now, the heat pulsing mode has been adopted as a solution to minimize the power dissipation, despite its adverse impact on the reliability of the microheater and the sensitive layer. It has been demonstrated that a rapid change in the microheater’s working temperature results in the formation of microcracks in the micro-hotplate, including the thin film sensitive layer, which dramatically reduces the reliability and lifetime of the device [3,4,5].
- The use of silicon as a substrate material allows for an integration of micro-electro-mechanical systems (MEMS) with complementary metal oxide semiconductor (CMOS) structures, which permits the use of robust materials for the microheater, thus improving the sensor reliability. This approach is highly challenging since the typically high temperatures associated with sensor fabrication negatively influence the front end of line devices and the metalization [6].
- The SMO gas sensor has the highest potential compared to other types of gas sensors available on the market, especially in terms of fabrication cost, footprint, sensitivity, and response time [7]. These features fulfill almost all the key specifications of the gas sensor with regard to portable device integration. Nevertheless, the poor selectivity and temporal drift of the SMO gas sensor is still hampering full practical integration [8,9,10].
- The functioning principle of gas sensors based on metal oxide films depends on heating the sensing layer to a temperature between 250 °C and 450 °C in order to enable a reaction between the sensitive material and ambient gases, as well as to allow for a full recovery of the signal after detection [11]. Minimizing this operating temperature, while having a good sensing response characteristic, is highly desired. A low temperature leads to a reduction in the overall power consumption and an improvement in the sensor reliability. Note that most issues related to the SMO gas sensor originate from its high operating temperature.

In this study, we fabricate Pt-doped SnO_2_ thin films on two different micro-hotplates in order to assess the best-performing sensor in terms of power consumption and baseline stability. Thereafter, we investigate the sensing characteristics toward formaldehyde and ethanol, including sensitivity and reproducibility, for both sensors. The aim of this work is to enhance the sensor sensitivity towards formaldehyde and ethanol, as well as to improve the baseline stability of the gas sensor response at low power consumption. For this, a novel design was conceived of, which aims to optimize the temperature uniformity over the active area, while keeping the power consumption at a minimum. To investigate the temperature behavior of the microheater and its impact on the micro-hotplate during operation, thermo-mechanical simulations and experimental characterizations were conducted. As the presented sensors are intended to be used in smartphones, the performance of the sensors is evaluated when operating in heat pulsing mode for several days, as well as in the presence of a rubber smartphone case in the vicinity of the gas sensor.

## 2. Materials and Methods

SMO gas sensors are the most investigated type of sensor among the gas sensor family since they can be used in many applications thanks to several outstanding features, mentioned in the previous section [12,13]. Amongst different metal oxides, SnO_2_ was proven to exhibit all of the features required for a high performing gas sensor [14]. Its enhanced ability to adsorb oxygen on its surface increases its sensitivity towards a variety of reducing gases [15]. In order to enhance the gas sensor response to VOCs, noble metal additives, and particularly platinum nanoparticles, have been sputtered onto the surface of the SnO_2_ thin film sensitive layer [16]. Thanks to platinum’s catalytic effect, VOCs in trace amounts down to concentrations of hundreds of ppb can be detected with good sensing response characteristics.

The microheater is one of the key components of the SMO gas sensor, as it defines several aspects of the sensor’s performance, such as power consumption, sensitivity, and selectivity [17]. To heat the microheater to elevated temperatures, as required for SMO sensing, joule heating is utilized due to its simplicity in implementation. In this study, tantalum aluminum (TaAl) has been chosen for the microheater material due to its ability to retain its mechanical strength at high operating temperatures and for its low temperature coefficient of resistance (TCR), which is −100 ppm/°C [18]. These properties make this material very promising compared to platinum, a commonly-used material as a micro-heater. A positive TCR increases the number of hotspots on the microheater [19], thus influencing the thermal performance of the gas sensor. Furthermore, the microheaters with high TCR, powered by the ASIC, require additional algorithms to keep the temperature within a desired range during operation. The output voltage and the input resistance of the ASIC are fixed within a specific range. One of the goals of this study is to enhance the temperature uniformity over the active area and to minimize the power consumption, while making the sensor compatible with application-specific integrated circuit (ASIC) features. For this, we adopt a novel design, the so-called composite heater, fabricated on a porous suspended membrane to minimize the thermal mass, as well as the thermal leaks from the heated area to the substrate.

### 2.1. Composite Heater

**Figure 1:**
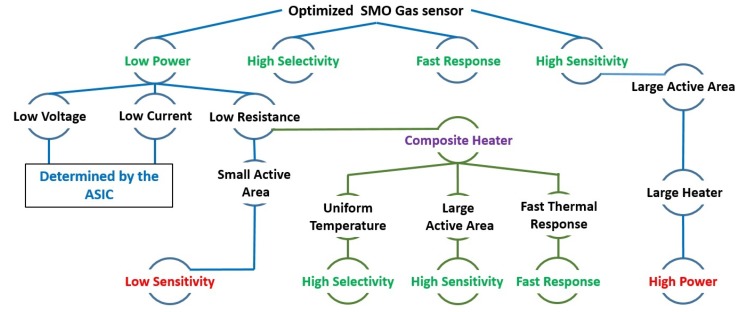
Limitations and required elements to enhance the sensing capability and performance of SMO gas sensors.

To improve the sensitivity of the SMO gas sensors, the heated area of the sensitive layer must be increased, since the chemical reactions involved are directly related to the amount of heated surface area in contact with the environment [20]. However, due to the high-temperature requirement, a large heated area means a larger heater and an increased power dissipation. The power consumption depends on the microheater resistance and gives rise to a compromise between a large heated area and a small resistance to reduce the power, as shown in Figure 1. The novel composite heater design presented here allows heating a large surface at a uniform temperature while having a small resistance, which results in a reduced power consumption. In addition, a small heater enables a very fast thermal response time, allowing the sensor to operate in short-pulsing mode and with a fast response time. With this novel microheater structure, the heater resistance is reduced, while the heated area is considerably increased, which improves the sensitivity of the device without a significant increase in power.

### 2.2. Fabrication and Design

**Figure 2:**
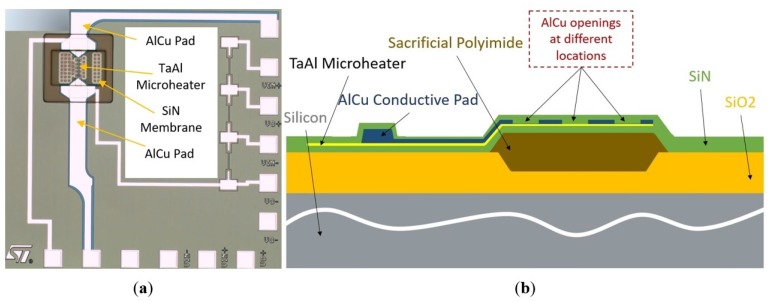
Structure of the SMO gas sensor. (**a**) Top view of the initial D02 design. (**b**) Cross-section of the novel composite heater design with the AlCu openings. To form the gas sensor membrane, the sacrificial polyimide was etched as the last step in the fabrication sequence using selective plasma etching in order to achieve low power consumption and good thermal isolation.

The composite heater was fabricated by etching openings in the AlCu conductive pads of an existing design, the so-called D02 design (Figure 2a), published previously [18]. With the openings, we create a succession of resistances, which are able to concentrate the heating of the sensitive material to desired locations. In the process flow, this etching step appears after the deposition of the TaAl microheater and the AlCu conductive pads, as shown in Figure 2b and described in detail in Ref. [18]. The total resistance of the heater must be between 50 Ω and 150 Ω in order to match the ASIC’s features.

**Figure 3:**
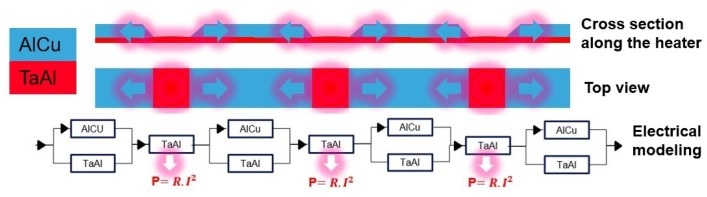
Cross-section view and top view of the composite heater along with its electrical modeling.

**Figure 4:**
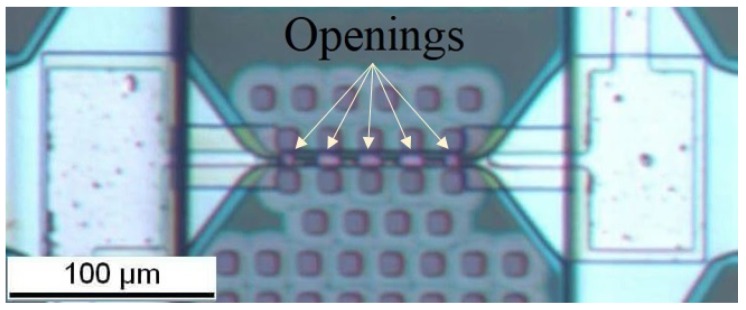
Top view of the fabricated composite heater with 5 openings. The three openings at the center have a length of 10 µm and a width of 6 µm, while the two openings at the extremities have a length and width of 6 µm.

The sheet resistance of TaAl is higher than that of AlCu, which are 13.5 Ω/sq and 0.025 Ω/sq at room temperature, respectively. Etching three openings in the AlCu with the same sizes leads to the creation of three identical resistances. Thus, when these three serial resistances operate under the same applied current, they provide the same temperature (Figure 3). It should be noted that, if the resistances are close to each other, the temperature profile of each resistance can change. The high thermal conductivity of the SiN membrane and the electrically-insulating layer, in addition to the AlCu conductive pads, allow for the heat to pass through the heater and then concentrate the high temperature at its center. To further improve the temperature uniformity over the entire microheater, two further openings with a reduced size, with respect to the openings at the center, are etched at the extremities of the microheater in such a way as to compensate the heat losses at the corners of the microheater (Figure 4).

In order to reduce the current density accumulation between TaAl and AlCu in each opening, a slope of 45° is etched at the end of each AlCu conductive pad. This additional fabrication step allows reducing the electrical losses and avoids breaking connection lines. Thereby, the power consumption is further decreased, and the reliability of the device is improved [21].

## 3. Results

The Pt-doped SnO_2_ thin films were deposited on two different micro-hotplates in order to assess the most efficient hotplate in terms of power consumption, temperature distribution, and mechanical stability. Thereafter, the sensing characteristics towards VOCs such as formaldehyde and ethanol were investigated at different concentrations. The effects of gas sensor operation in heat pulsing mode and near a rubber smartphone case on the sensing performance are highlighted.

### 3.1. Device Simulations

#### 3.1.1. Thermal Analyses

The sensors were designed and simulated using the COMSOL Multiphysics finite element software [22], where the boundary conditions and initial material temperatures were set to 25 °C for all materials of the gas sensors and in the ambient environment. The TaAl TCR and the temperature of the substrate bottom were fixed at −100 ppm/°C and 25 °C, respectively. To calculate the heat losses to the air by conduction and convection, while taking fluid motion into account, 233.46 W m^−2^K^−1^ was used as the heat transfer coefficient, when the temperature of the top and bottom surface (heated area) of the membrane reached 300 °C [18]. It should be pointed out that the convection losses are insignificant at medium-to-low temperatures for small micro-hotplates [23]. Additionally, radiation losses are insignificant and therefore not considered for temperatures below 400 °C [24].

**Figure 5:**
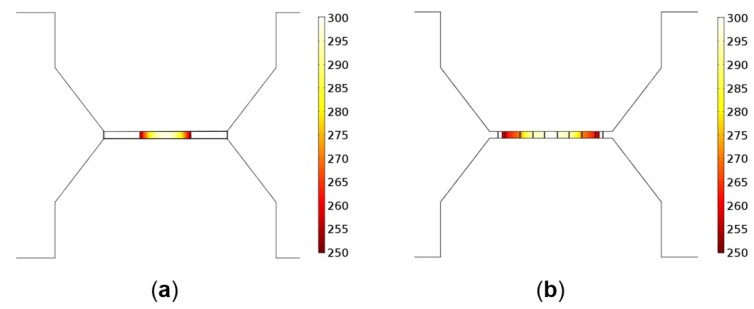
Temperature distribution (°C) over the active area of (**a**) the initial D02 design and (**b**) the novel composite heater design.

Figure 5b shows the temperature distribution over the composite heater design, where a high uniformity in temperature distribution over the active region can be observed. This is a significant improvement compared to the D02 design, depicted in Figure 5a. The improved uniformity in temperature has been achieved in the composite heater design thanks to the high thermal conductivity of the AlCu conductive pads and the SiN membrane and insulating layer. The heating process is no longer activated by the microheater alone, but also by the AlCu and SiN. Therefore, even though the total resistance is reduced, a larger overall active sensing area is achieved by separating the heating locations.

#### 3.1.2. Mechanical Analysis

The thermal-induced stresses for both designs were simulated at various working temperatures. The time-temperature variations along with the difference in the thermal expansion coefficients between Si_3_N_4_, TaAl, and AlCu generated thermally-induced stress and strain, leading to undesirable structural deformations. At room temperature, the residual deflection after fabrication of both membranes in the active region was around 50 nm and 20 nm, for the composite heater and D02 designs, respectively. The average residual stress present in the membrane of the composite heater to produce this deflection was −45.9 MPa, while the D02 had −44.2 MPa to produce a 20-nm deflection. These stress values are within the acceptable range of |$\sigma_{residual}$| < 0.1 GPa, as reported by Rossi et al. [25]. Note that the stress and deformation profile of the membrane may change at different operating temperatures.

**Figure 6:**
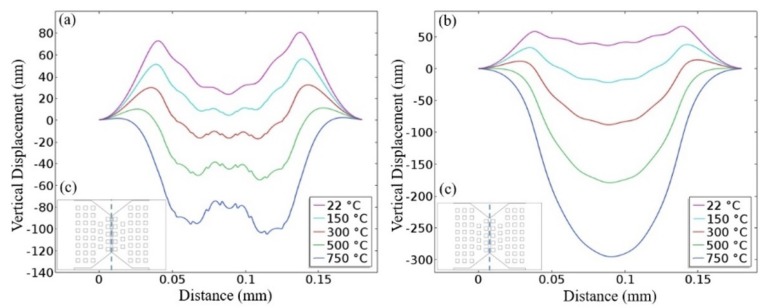
Simulated vertical deformation of the membranes at different operating temperatures. (**a**) Composite heater design. (**b**) D02 design. (**c**) Cut-line of the inset representing the location of the results displayed in (**a**,**b**).

Figure 6 shows the simulated displacement profile of the membrane of the composite heater and D02 designs as a function of the operating temperature. At low temperatures, only the thermal expansion of the stacked layers impacted the deformation profile of the membrane, causing a downwards deflection. However, at medium-to-high temperatures, the membrane of the composite heater bent upwards in the active region. This behavior was mainly caused by the bimetallic effect, as the heated area of the composite heater contained two metals, TaAl and AlCu. From experimental measurements, the composite heater failed at temperatures around 500 °C, while the D02 heater failed at 720 °C, as a result of crack build-up in the heater [18]. The average stresses required to rupture the heater were −103.9 MPa and −91.3 MPa, for the composite and D02 designs, respectively. It should be noted that the thickness of the SnO_2_ thin film was around 100 nm, which means a slight deformation, even at the nano-scale, in the active region may impact the sensing characteristics of the sensor due to crack formation at the surface of the sensitive layer. Therefore, operation in heat pulsing mode can result in a dramatically-reduced lifetime of the sensor.

### 3.2. Sensing Performance

#### 3.2.1. Sensing Characteristics for VOCs

Both sensors have been characterized in a micro-chamber with the aim to test and study their performances under exposure to formaldehyde and ethanol. The relative humidity and the gas flow rate were fixed at 55% and 300 sccm, respectively. The sensors were exposed to increasing formaldehyde and ethanol concentrations from 100 ppb–5 ppm (0.1, 0.5, 1, 2.5, 5 ppm) for a duration of 1 min. This experiment was performed once. To control the surrounding temperature and humidity, the micro-chamber was placed in an environmental chamber. The gas concentrations inside the micro-chamber were controlled and monitored using several valves, connected to a PC. In order to measure the resistance of the thin film sensitive layers, Keithley 2450 SourceMeters were used at a bias current of 100 nA.

**Figure 7:**
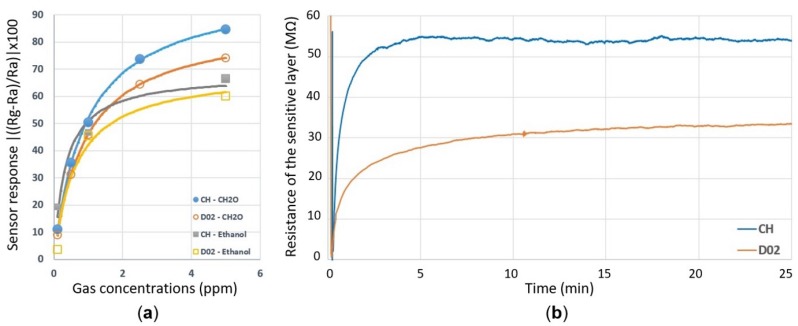
Sensing characteristics of the composite heater (CH) and the D02 design operating at 10.5 mW. (**a**) The gas sensor response towards formaldehyde and ethanol gases at concentrations in the ppb and ppm range. The curves in (**a**) represent the equations depicted in Table 1 to fit the data. (**b**) Baseline response of the two sensors during operation for 25 min.

Figure 7a shows the sensor responses of the composite heater design and the D02 design towards formaldehyde and ethanol at varying concentrations. Each sensor was operating at a power of 10.5 mW in order to heat up the layer to the required elevated temperature. An improvement in the sensitivity of the Pt-doped SnO_2_ thin film towards VOCs was achieved with the composite heater when compared to the original D02 design. The stability of the baseline was also tested for an extended period, as shown in Figure 7b. The composite heater design showed a highly stable baseline after about 5 min, while the baseline of D02 still showed a drift even after 25 min of operation.

**Figure 8:**
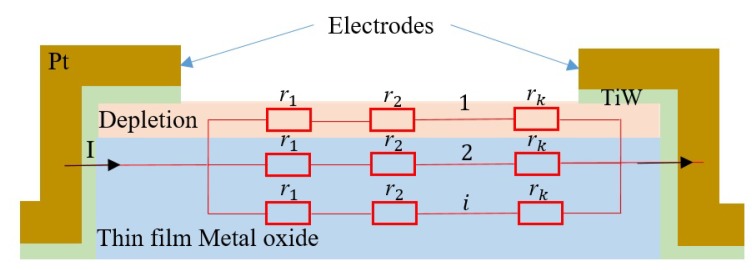
Device configurations and the corresponding equivalent circuit to investigate the adsorption of the metal oxide film in SMO gas sensors.

A simple model based on the Langmuir adsorption model was used to fit the experimental sensitivity data as a function of the gas concentration [26]. This model involves only the equilibrium state. It is based on the following characteristic assumptions: equivalence of all adsorption sites and surface uniformity, no interactions between adsorbed molecules, and adsorption of a single layer (monolayer coverage). As shown in Figure 8, the overall resistance of the sensing film can be considered as *i* resistances of *R* in parallel, where each *R* is composed of *k* resistances of *r* in series. Here, *R*, *r*, and *i* represent the resistance of a layer, the resistance of a site, and the number of conduction paths, respectively, while *k* is the number of active sites in a monolayer. Thus, the resistance *R* is given by:

(1) $$R=k\cdot \beta \cdot r_{1}+k\left(1-\beta \right)r_{0},$$

where $r_{0}$ is the resistance of a vacant site, while $r_{1}$ is the resistance of an occupied site; β is the site coverage of adsorption. According to the Langmuir adsorption model, β can be expressed as:

(2) $$\beta =\frac{T_{k}\cdot C}{1+T_{k}\cdot C},$$

where $T_{k}$ is the adsorption equilibrium constant and *C* the concentration of the analyte. Combining (1) and (2) gives the expression for the sensing response:

(3) $$S=\left(r_{1}-r_{0}\right)\frac{k}{i}\frac{T_{k}\cdot C}{1+T_{k}\cdot C};$$

as the electrodes were deposited on the top of the sensitive layer, only the resistance of the depletion region was measured. Therefore, (3) can be written as follows:

(4) $$S=a\frac{b\cdot C}{1+b\cdot C},$$

given that

(5) $$a=(r_{1}-r_{0})k$$

and

(6) $$b=T_{k},$$

where *a* and *b* are fitting parameters. Their numerical values are depicted in Table 1.

**Figure 9:**
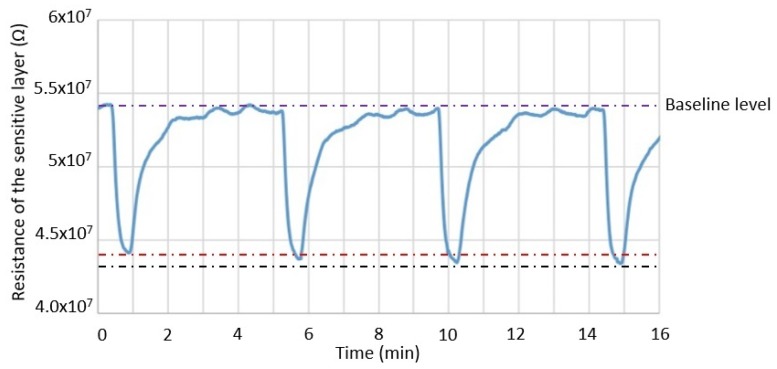
Response reproducibility of the composite heater towards 100 ppb of ethanol while the sensor is operating at 10.5 mW. The red and black lines represent the lowest and highest resistivity observed during exposure to 0.1 ppm of ethanol, respectively.

In order to investigate the reproducibility of the composite heater design towards 100 ppb of ethanol, the test gas flow was turned on for 30 s and then turned off for 4 min. This sequence was repeated four times while the sensor was operating at a power consumption of 10.5 mW. A very small difference in the sensitivity between the four tests was observed (Figure 9). The sensitivity measured in the first sequence was 18.63%, while in the last one, the sensitivity was 19.48%; a variation of only 0.84%. The reproducibility of the sensor might be impacted by the inherent variability of the valves used for gas injection at concentrations below 1 ppm.

**Figure 10:**
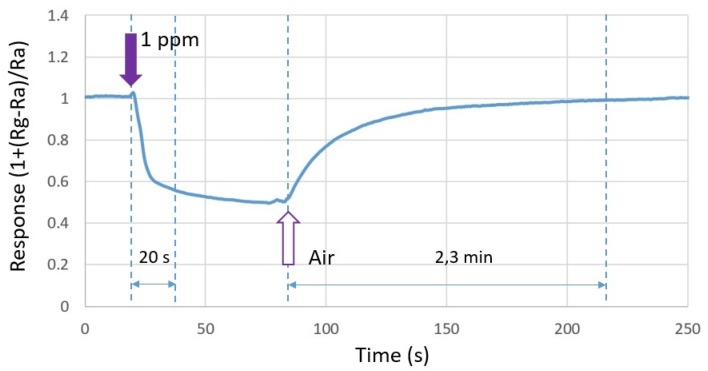
Response time and recovery time of the composite heater design with respect to 1 ppm of formaldehyde.

Figure 10 shows the response time and recovery time of the composite heater design when exposed to 1 ppm of formaldehyde. The response time was in the range of 18–20 s, which is very satisfactory for applications dedicated to air quality monitoring. It is defined as the time required for the conductance to change to 90% of its final value. The recovery time was about 2.3 min. To accelerate the recovery of the sensor, one simple solution is to further elevate the operating temperature until the sensor response is fully recovered to its baseline [27]. At this stage, the sensor is ready for further use.

#### 3.2.2. Dynamic Response

The composite heater design was integrated with the ASIC and then assembled with a test board to characterize its sensing performance when operating in heat pulsing mode. The heater was powered on for only a fraction of the time to reduce the average power consumption. An on-off time of 1:24 was used in this study, which gives an average power consumption of 0.42 mW. To configure the repetitive pulse scenario and extract the measured data by the composite heater sensor, as well as to power the system, the test board was connected to a PC using a USB cable. An application was developed specifically for this task, allowing us to establish a connection with the test board and to observe real-time changes in the resistance of the sensitive layer.

**Figure 11:**
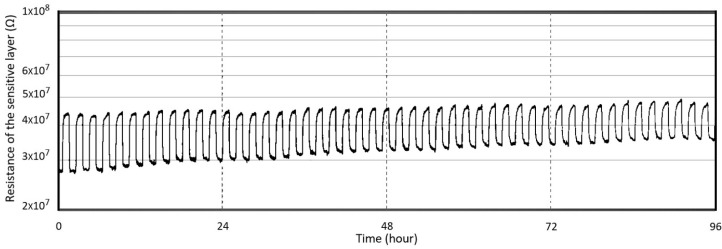
Response of the composite heater sensor towards 20 ppm of formaldehyde when operating in heat pulsing mode for four days.

The characterization of the sensing performance of the ASIC-integrated composite heater design was carried out in the same environment as the previous characterizations. A concentration of 20 ppm of formaldehyde was injected into the micro-chamber for 1 h every 2 h. The measurements of the sensor response for four sequential days is depicted in Figure 11. During this period, a gradual decrease in the sensitivity was observed as the operating cycles increased. The thermal stress created by the temperature cycling, as revealed by thermo-mechanical simulations, may cause the thin film to crack, which eventually impacts the sensing performance of the sensor [4]. These cracks provide short-circuit pathways for sensing gases, moisture, and oxygen. Moisture diffusion through the pathways is the primary explanation for the deterioration of the sensitivity of the tin oxide gas sensors [3]. A drift in the resistivity in the Pt-doped SnO_2_ sensitive thin film has also been noted. The most likely reason for this drift is the change in the surface microstructure [4]. However, another reason could be the build-up of strain in the sensing film. A strained film will have a different baseline resistivity than the unstrained alternative.

#### 3.2.3. Effects of Smartphone Cases

**Figure 12:**
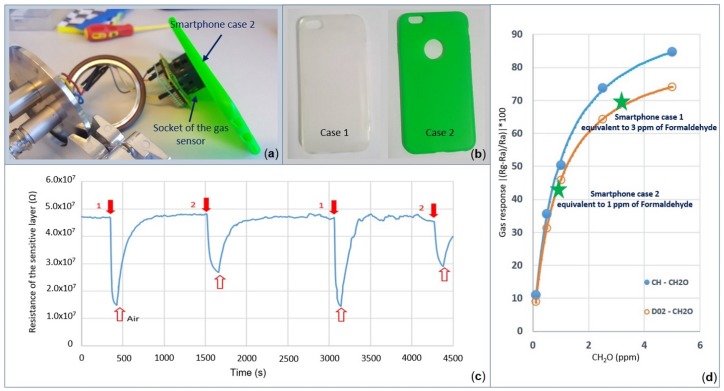
Response of the gas sensor while operating near the rubber smartphone cases in open air. (**a**) Characterization setup. (**b**) Two rubber smartphone cases used in this study. (**c**) Resistance of the sensitive layer of the D02 gas sensor in the presence of rubber smartphone cases. (**d**) Equivalent response of the smartphone covers when equated to formaldehyde.

**Figure 13:**
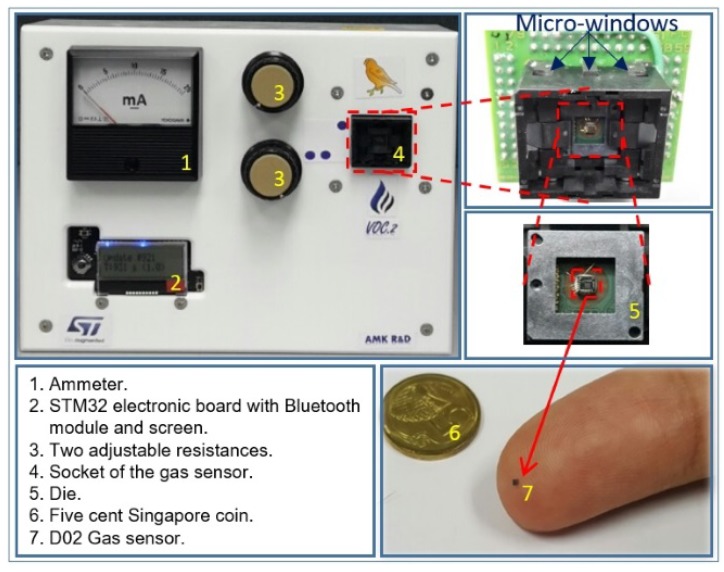
Canary board along with its different components.

In this section, two simple methods to characterize the sensing performance of the gas sensors, while operating near rubber smartphone cases, are highlighted. For the first experiment, two smartphone covers for an iPhone 6, Case 1 and Case 2 from Figure 12b, are used to evaluate the selectivity of the gas sensor in air. Each smartphone cover is placed for at least one minute in front of the socket while leaving some space between the gas sensor D02 and the smartphone case (Figure 12a). Note that the three micro-windows on top of the socket allowed fresh air to enter inside the socket continuously during the test (Figure 13). It was observed that the resistivity of the sensitive layer reduced dramatically in the presence of the smartphone case. The sensor response exhibited the same behavior when sensing reducing gases. The measured responses of the gas sensor in the presence of Smartphone Case 1 and Smartphone Case 2 were found to be equivalent to the response of the gas sensor D02 when, sensing 3 ppm and 1 ppm of formaldehyde, respectively (Figure 12d). After removing the smartphone cover, the signal output returned to its initial state.

Due to the difficulty in ensuring that the gas sensor and the rubber smartphone case can physically fit in the same micro-chamber, where the air composition is well controlled, an embedded system was conceived of and fabricated for this purpose. The so-called canary board uses an STM32 electronic board on which the gas sensor, Bluetooth module, temperature sensor, and LCD display are connected. The power was supplied using an external 5-V battery, which was further adjustable by two variable resistors; in this system, one can both visualize the current and measure the voltage. All components were included in a plastic nomad box, as shown in Figure 13. Data, such as the temperature and the resistance of the sensitive layer of the gas sensor, were sent to a computer for post-processing and visualization using a Bluetooth connection. Afterwards, one can visualize the sensor response simultaneously on a graph using a Windows application developed using the Visual Studio development environment.

**Figure 14:**
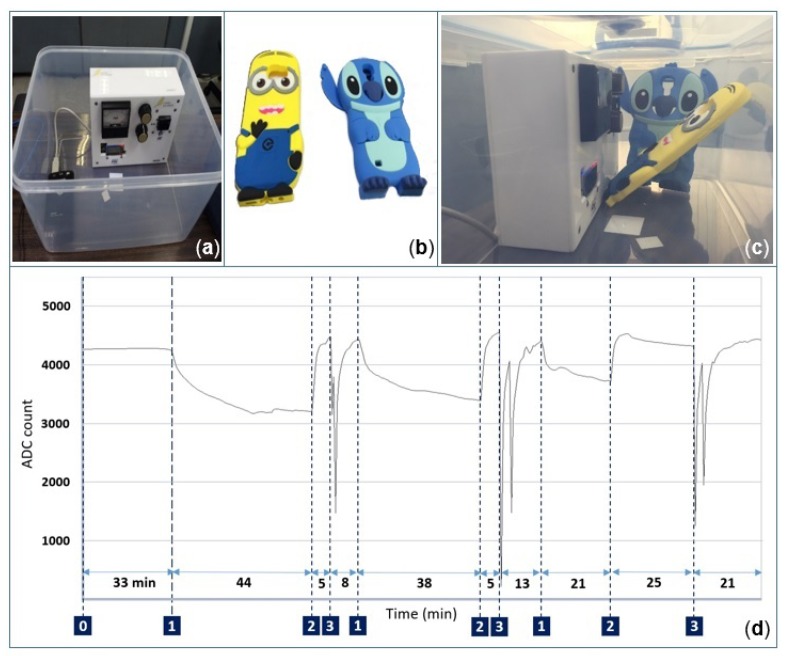
Response of the gas sensor while operating in a closed chamber with and without smartphone covers. (**a**) Two-liter chamber. (**b**) Rubber smartphone cases for Samsung S5 and S6. (**c**) Canary board along with smartphone cases in the same closed chamber. (**d**) Measured resistance of the sensitive layer (not calibrated) during the experiment.

The second experiment was performed in a confined environment using a Bluetooth connection between the canary board and a PC, avoiding the need for a USB cable to collect data. To further support the previous experimental results, the canary board and two new smartphone covers for Samsung S5 and S6 smartphones were placed together in a two-liter chamber (Figure 14c). The experiment was performed as follows; note that the numbers on the left correspond to the experimental stages of the resistance of the sensitive layer (not calibrated) labeled in Figure 14d:

0 During the first 33 min, the gas sensor operated at 12 mW in a closed box. Note that only the canary board with its external battery was in the box.
1 The box was opened, and both smartphone covers were placed inside the box; afterwards, the chamber was closed again for 44 min.
2 The smartphone covers were removed from the box.
3 Specially-denatured (SD) alcohol-40 was injected two times with random concentrations near the gas sensor while the box was opened. This step was introduced to check whether the sensor was still working normally after the other tests were performed.

The aforementioned sequence was repeated three times with varying durations. It was found that each time the smartphone covers were introduced into the box, the baseline decreased gradually over time, as can be noted in Figure 14d. This is in good agreement with the previous experiment. In addition, an anomalous response was observed during the last 40 min, when the baseline started drifting in the downside instead of the upside. It is possible that the particles released by the smartphone covers contaminated the sensitive layer of the gas sensor.

## 4. Discussion

In order to optimize and improve the performance of the previous D02 SMO gas sensor design, while making the sensor compatible with ASIC features, a novel sensor was designed, fabricated, and characterized. The characteristics of both designs in terms of sensing capability of the sensitive layer and the thermo-mechanical behavior of the micro-hotplate have been qualitatively compared. The response of the composite heater towards formaldehyde and ethanol was found to be significantly improved using the new composite design, when compared to the D02 design at 10.5 mW of power consumption. Concerning the response reproducibility, a minimal deviation was observed. The baseline responses have also been tested for a period of 25 min. The composite heater showed a highly stable baseline compared to that of the D02 design. Regarding the response and recovery times of the sensors, both designs demonstrated satisfactory results. The response time was in the range of 18–20 s, while the recovery time was about 2.3 min. As the presented designs were conceived of for mobile applications, the composite heater design was integrated with the ASIC and then assembled with a test board to characterize its sensing performance when operating in heat pulsing mode. During this test, a gradual decrease in the sensitivity was observed as the number of operating cycles increased. In addition, a drift in the resistivity of the Pt-doped SnO_2_ sensitive thin film was observed. This sensitive layer was found to be affected by particles released from the rubber smartphone case, thereby reducing the resistivity of the sensitive layer dramatically in a manner analogous to the effects of reducing gases.

## 5. Conclusions

We have enhanced the sensing characteristics of the previous D02 SMO gas sensor design by changing the structure of its microheater. The novel structure was fabricated by etching openings in the AlCu conductive pads of the D02 design. With the openings, we created multiple resistances that were able to concentrate the heating to desired locations. This way, the overall resistance of the microheater was reduced from 290 Ω to less than 150 Ω, while the active area was significantly enlarged. Reducing the microheater resistance to the range of 50 Ω–150 Ω allowed integrating the sensor with the ASIC. The composite heater was stacked and subsequently connected to the ASIC with the aid of glue and bonding wires, leading to electrical contact measurement of the sensitive layer resistance and driving the heater, as well as data processing. By being compatible with the ASIC features, the SMO gas sensor presented here can be integrated with other MEMS sensors and actuators in the same chip, such as a pressure and humidity sensor. As a proof of concept, this device is used to create a formaldehyde and ethanol gas sensor, but it can be used for a wide variety of sensing applications, namely food and air quality monitoring, sensor networks, RFID tags, and medical applications. In order to make the presented sensor suitable for wearable devices, especially for mobile applications, one must be selective towards particles released by smartphone cases, and its power consumption must be further decreased without using a heat pulse mode.

## Figures and Tables

**Table 1:** Summary of the sensing characteristics of both designs, shown in Figure 7a. The equation column calculated using the Langmuir isotherm model shows the best-fit lines for the sensitivity of the gas sensors (solid lines in Figure 7a).

Reacting Gas/Gas Sensor Design	Equation	Concentration
Formaldehyde/CH	$100.59\times \frac{1.067\times C}{1+1.067\times C}$	0.1 ppm–5 ppm
Formaldehyde/D02	$87.504\times \frac{1.111\times C}{1+1.111\times C}$	0.1 ppm–5 ppm
Ethanol/CH	$68.259\times \frac{2.943\times C}{1+2.943\times C}$	0.1 ppm–5 ppm
Ethanol/D02	$69.475\times \frac{1.541\times C}{1+1.541\times C}$	0.1 ppm–5 ppm

## Abbreviations

The following abbreviations are used in this manuscript:

SMO	Semiconducting metal oxide
VOCs	Volatile organic compounds
WHO	World Health Organization
TCR	Temperature coefficient of resistance
ASIC	Application-specific integrated circuit
CH	Composite heater
MEMS	Micro-electro-mechanical systems
CMOS	Complementary metal-oxide-semiconductor
SD	Specially denatured

## References

[1] MarketsandMarkets (2018). Gas Sensors Market Worth 1297.6 Million USD by 2023. https://www.marketsandmarkets.com/PressReleases/gas-sensor.asp.

[2] World Health Organization (2018). 9 Out of 10 People Worldwide Breathe Polluted Air, But More Countries Are Taking Action. http://www.who.int/news-room/detail/02-05-2018-9-out-of-10-people-worldwide-breathe-polluted-air-but-more-countries-are-taking-action.

[3] Tang Z., Chan P.C.H., Sharma R.K., Yan G., Hsing I.-M., Sin J.K.O. (2001). Investigation and control of microcracks in tin oxide gas sensing thin-films. Sens. Actuators B Chem..

[4] Sharma R.K., Chan P.C.H., Tang Z., Yan G., Hsing I.-M., Sin J.K.O. (2001). Investigation of stability and reliability of tin oxide thin-film for integrated micro machined gas sensor devices. Sens. Actuators B Chem..

[5] Prasad M. (2015). Design, development and reliability testing of a low power bridge-type micromachined hotplate. Microelectron. Reliab..

[6] Filipovic L., Lahlalia A. (2018). System-on-chip sensor integration in advanced CMOS technology. ECS Trans..

[7] Yi S., Tian S., Zeng D., Xu K., Peng X., Wang Z.S., Xie C. (2014). A novel approach to fabricate metal oxide nanowire-like networks based coplanar gas sensors array for enhanced selectivity. Sens. Actuators B Chem..

[8] Romain A.C., Nicolas J. (2010). Long term stability of metal oxide-based gas sensors for e-nose environmental applications: An overview. Sens. Actuators B Chem..

[9] Padilla M., Fonollosa J., Marco S. (2013). Improving the robustness of odor sensing systems by multivariate signal processing. Human Olfactory Displays and Interfaces: Odor Sensing and Presentation.

[10] Padilla M., Perera A., Montoliu I., Chaudry A., Persaud K., Marco S. (2010). Drift compensation of gas sensor array data by orthogonal signal correction. Chemom. Intell. Lab. Syst..

[11] Wang C., Yin L., Zhang L., Xiang D., Gao R. (2010). Metal oxide gas sensors: Sensitivity and influencing factors. Sensors.

[12] Kalantar-Zadeh K., Yao C.K., Berean K.J., Ha N., Ou J.Z., Ward S.A., Pillai N., Hill J., Cottrell J.J., Dunshea F.R. (2016). Intestinal gas capsules: A proof-of-concept demonstration. Gastroenterology.

[13] Konduru T., Rains G.C., Li C. (2015). A customized metal oxide semiconductor-based gas sensor array for onion quality evaluation: System development and characterization. Sensors.

[14] Patil G.E., Kajale D.D., Gaikwad V.B., Jain G.H. (2012). Spray pyrolysis deposition of nanostructured tin oxide thin films. ISRN Nanotechnol..

[15] Haridas D., Sreenivas K., Gupta V. (2008). Improved response characteristics of SnO_2_ thin film loaded with nanoscale catalysts for LPG detection. Sens. Actuators B Chem..

[16] Kim B.Y., Cho J.S., Yoon J.W., Na C.W., Lee C.S., Ahn J.H., Kang Y.C., Lee J.H. (2016). Extremely sensitive ethanol sensor using Pt-doped SnO2 hollow nanospheres prepared by Kirkendall diffusion. Sens. Actuators B Chem..

[17] Gharesi M., Ansari M. (2016). Tin oxide microheater for chemical sensors. IOP Conf. Ser. Mater. Sci. Eng..

[18] Lahlalia A., Le Neel O., Shankar R., Kam S.Y., Filipovic L. (2018). Electro-thermal simulation & characterization of a microheater for SMO gas sensors. J. Microelectromech. Syst..

[19] Bhattacharyya P. (2014). Technological journey towards reliable microheater development for MEMS gas sensors: A review. IEEE Trans. Device Mater. Reliab..

[20] Sharma A., Tomar M., Gupta V. (2013). Enhanced response characteristics of SnO_2_ thin film based NO_2_ gas sensor integrated with nanoscaled metal oxide clusters. Sens. Actuators B Chem..

[21] Lahlalia A., Filipovic L., Selberherr S. (2018). Modeling and simulation of novel semiconducting metal oxide gas sensors for wearable devices. IEEE Sens. J..

[22] (2016). COMSOL Multiphysics.

[23] Götz A. (1997). Integrated Microsensors for Microsystems with Application in Biomedicine and Environmental Control. Ph.D. Thesis.

[24] Simon I., Bârsan N., Bauer M., Weimar U. (2001). Micromachined metal oxide gas sensors: Opportunities to improve sensor performance. Sens. Actuators B Chem..

[25] Rossi C., Temple-Boyer P., Estève D. (1998). Realization and performance of thin SiO_2_/SiN_*x*_ membrane for microheater applications. Sens. Actuators A Phys..

[26] Lin C.W., Hwang B.J., Lee C.R. (1999). Characteristics and sensing behavior of electrochemically codeposited polypyrrole-poly (vinyl alcohol) thin film exposed to ethanol vapors. J. Appl. Polym. Sci..

[27] Magno M., Jelicic V., Chikkadi K., Roman C., Hierold C., Bilas V., Benini L. (2016). Low-power gas sensing using single walled Carbon nano tubes in wearable devices. IEEE Sens. J..

